# Circulating miR-183-5p levels are positively associated with the presence and severity of coronary artery disease

**DOI:** 10.3389/fcvm.2023.1196348

**Published:** 2023-06-15

**Authors:** Dong Lv, Yanfu Guo, Li Zhang, Xia Li, Guangping Li

**Affiliations:** ^1^ Department of Cardiology, Tianjin Key Laboratory of Lonic-Molecular Function of Cardiovascular Disease, Tianjin Institute of Cardiology, The Second Hospital of Tianjin Medical University, Tianjin, China; ^2^ Department of Cardiology, Beijing Renhe Hospital, Beijing, China; ^3^ Department of Clinical Medicine, Graduate School of Jiamusi University, Heilongjiang, China; ^4^ Department of Cardiology, Hegang People’s Hospital, Heilongjiang, China; ^5^ Department of Clinical Medicine, Jiamusi University, Heilongjiang, China

**Keywords:** miR-183-5p, coronary artery disease, severity, Gensini score, association

## Abstract

**Background:**

Serum miR-183-5p levels are associated with carotid atherosclerosis, while less is known about the relationship between circulating miR-183-5p levels and stable coronary artery disease (CAD).

**Methods:**

In this cross-sectional study, consecutive patients with chest pain who underwent coronary angiograms from January 2022 to March 2022 at our center were enrolled. Those presenting acute coronary syndrome or had a prior CAD were excluded. Clinical presentations, laboratory parameters, and angiographic findings were collected. Serum miR-183-5p levels were measured using quantitative real-time polymerase chain reaction. CAD severity was displayed as the number of diseased vessels and further evaluated by the Gensini score system.

**Results:**

Overall, 135 patients (median age, 62.0 years; male, 52.6%) were included in the present study. Stable CAD was identified in 85.2% of the study population, with 45.9% having 1-vessel disease, 21.5% having 2-vessel disease, and 17.8% having 3-vessel or left main disease. Serum miR-183-5p levels were significantly increased in CAD patients with different severities than non-CAD patients (all adjusted *p* < 0.05). Serum miR-183-5p levels increased as tertiles of the Gensini score progressed (all adjusted *p* < 0.05). Importantly, serum miR-183-5p levels could predict the presence of CAD and 3-vessel or left main disease in the receiver operating characteristic curve analysis (both *p* < 0.01), and also in multivariate analysis adjusting for age, sex, body mass index, diabetes, hypersensitive-C-reactive protein (both *p* < 0.05).

**Conclusion:**

Serum miR-183-5p levels are independently and positively correlated with CAD presence and severity.

## Introduction

Coronary artery disease (CAD) remains a major cause of mortalities and morbidities worldwide (1). Approximately 11% of adults ≥ 45 years and 17% of adults ≥ 65 years are probably to have CAD, and around 800,000 suffer a myocardial infarction (MI) every year in the U.S. (2). In 2018, CAD mortality was 365,744 and MI mortality was 108,610 in the U.S. (2). These lead to a significant healthcare burden, expected to increase to >$177 billion by 2040 in the U.S. (3). Importantly, in-hospital mortality did not improve in patients with ST-segment elevation MI (STEMI) undergoing percutaneous coronary intervention (PCI) (4). Similarly, overall mortality of acute MI continued to increase since 2002 in both the urban and rural area of China. Given the increasing prevalence of CAD and its risk factors (e.g., advanced age, obesity), there is an unmet need to discover an optimal biomarker predicting the presence and severity of CAD.

MicroRNA (miRNA or miR) has been known to regulate gene expression at the post-transcriptional level (5). More than half of human protein-coding genes are estimated to be modulated by miRNA, given one miRNA can regulate the expression of several transcripts (6). Moreover, miRNA can influence cell proliferation, differentiation, and death in the circulatory system (7). Meanwhile, several circulating miRNAs have shown promising for early detection, severity evaluation, and outcome prediction of CAD (8).

miR-183-5p is already known as an oncomir, highly expressed in tumor tissues (9–11). Recently publications revealed that patients with carotid atherosclerosis had higher serum miR-183-5p levels compared with health individuals (12, 13). Meanwhile, elevated expression of circulating miR-183-5p was also detected in both patients with acute coronary syndrome (ACS) and non-ST-segment elevation MI (NSTEMI) (14, 15). Thus, circulating miR-183-5p could potentially be a promising biomarker for atherosclerotic and/or thrombotic disease. However, serum miR-183-5p levels have never been investigated in patients with stable CAD, the most common category of CAD. To fill this gap, we performed this cross-sectional study to examine the relationship between serum miR-183-5p levels and the presence of CAD, as well as the severity of CAD.

## Material and methods

### Study population and sample

We prospectively enrolled consecutive patients with chest pain who underwent invasive coronary angiograms to determine the presence of stable CAD from January 2022 to March 2022 at Beijing Renhe Hospital, Beijing, China. Patients were excluded from the present study if they had: (1) age < 18 or ≥ 80 years old; (2) history of established arteriosclerotic cardiovascular disease (ASCVD) or vascular revascularization; (3) ACS at this admission; (4) major organ failure (e.g., heart, liver, kidney); (5) active infectious disease, autoimmune diseases, and malignancy.

Fasting venous blood was collected for measuring plasma total triglyceride, total cholesterol, low-density lipoprotein cholesterol (LDL-C), glycosylated hemoglobin, creatinine, and hypersensitive-C-reactive protein (hs-CRP). The blood serum was isolated and stored at −80°C. We also collected the patient's clinical characteristics and risk factors for CAD. Body mass index (BMI) was calculated as weight divided by the square height. Echocardiography was used to evaluate cardiac function and structure using a GE ViVid E7 ultrasonography (GE Healthcare, USA). This study was approved by the Ethics Committee of Beijing Renhe Hospital (RH20220103), and performed following the Declaration of Helsinki. Written informed consent was obtained from all participants.

### Quantitative real-time polymerase chain reaction

Total RNA was extracted from the preserved serum samples using Trizol reagent (Invitrogen, USA). RNA (0.5 μg) was reverse transcribed with PrimeScript RT Reagent Kit (Takara, Japan). Then, quantitative real-time polymerase chain reaction (qRT-PCR) was performed using the CFX Real-Time PCR Detection System (Bio-Rad, USA). The thermocycling amplification protocol was as follows: denaturation at 95°C for 3 min, followed by 40 cycles of 95°C for 15 s and 30 s at 60°C. Expression of miR-183-5p was calculated based on the 2^−ΔΔCt^ method. The primer sequences were as follows: miR-183-5p, forward 5′-CGCGGTATGGCACTGGTAGA-3′, reverse 5′-AGTGCAGGGTCCGAGGTATTC-3′; U6 (internal control), forward 5′-CTCGCTTCGGCAGCACAT-3′, reverse 5′-TTTGCGTGTCATCCTTGCG-3′.

### Coronary angiography and intervention

Invasive coronary procedures and periprocedural management were performed by international guidelines (16). Briefly, diagnostic coronary angiography was performed by experienced interventionists, who were blind to the patient's serum miR-183-5p levels, using the transradial or transfemoral approach. The location and severity of each coronary artery stenosis were assessed by two independent interventionists, and discrepancies were solved with discussion.

CAD was defined as any major epicardial coronary stenosis ≥ 50%. Thus, patients were divided according to the number of diseased vessels: 0-vessel disease (i.e., non-CAD), 1-vessel disease, 2-vessel disease, 3-vessel or left main (LM) disease. In addition, we quantified the severity of CAD using the Gensini score system (17). Thus, in the current study, CAD severity was presented using either the different numbers of diseased vessels or the tertiles of Gensini score. Finally, the decision of coronary intervention was made at the discretion of the interventionist.

### Statistical analysis

Continuous variables were shown as median (interquartile range), and compared using the Kruskal-Wallis H test. The Bonferroni correction was used to adjust the *p* value in multiple-comparison analysis. Categorical variables were expressed as numbers (percentages), and compared using the Chi-square test or Fisher's exact test. To investigate the relationship between serum miR-183-5p levels and CAD severity, we firstly compared serum miR-183-5p levels among a varied number of diseased vessels and tertiles of Gensini score (low tertile, <15.3; middle tertile, 15.3-30.0; high tertile, >30.0). Then, this relationship was examined using Spearman's correlation analysis, shown as *r* and its 95% confidence interval (CI). Meanwhile, we also determined different CAD severity and extent across the tertile of serum miR-183-5p levels. The receiver operating characteristic (ROC) curve was performed to explore the diagnostic value of the serum miR-183-5p level for the presence and severity of CAD. The area under the ROC curve (AUC) with its 95% CI was used to measure the predictive ability of miR-183-5p. The Youden index was used to determine the optimal diagnostic threshold of miR-183-5p, and its corresponding sensitivity and specificity. Finally, the predictive value of miR-183-5p on the presence and severity of CAD was investigated in a multivariate model using logistical regression analysis, in which co-variables included age, sex, BMI, and those with a *p -v*alue < 0.1 in the univariate analysis. A two-sided *p-v*alue < 0.05 was considered statistically significant. All statistical analyzes were conducted using SPSS 20.0 software (IBM, Armonk, New York).

## Results

### Patient characteristics and angiographic findings

Overall, 135 patients (median age, 62.0 years; male, 52.6%) were included in the present study, who underwent invasive coronary artery angiogram to determine whether CAD was the origin of chest pain (Table 1). Hypertension (71.9%), hyperlipidemia (54.1%) and, current smoking (45.2%) were common risk factors for CAD. Notably, baseline total cholesterol (median, 5.1 mmol/L) and LDL-C (median, 3.2 mmol/L) levels were relatively high (Table 1), resulting in 92.6% of patients prescribed statins (Table 2). In addition, left ventricular systolic function (median left ventricular ejection fraction, 66.0%) and structure (median left ventricular end-diastolic diameter, 46.0 mm) were preserved (Table 1). Importantly, baseline characteristics were comparable across different CAD severities (*p* > 0.05), except for hs-CRP levels (*p* = 0.005). In particular, higher hs-CRP levels were observed in 3-vessel or LM disease (adjusted *p* < 0.05) and 2-vessel disease subgroups (adjusted *p* < 0.05), compared to non-CAD patients (Table 1).

**Table 1:** Baseline characteristics of the study population.

	Overall *N* = 135	Non-CAD *N* = 20	CAD			*p*-value
			1-vessel disease *N* = 62	2-vessel disease *N* = 29	3-vessel/LM disease *N* = 24	
**Demographics**						
Age, years	62.0 (56.0–69.0)	58.5 (53.5–66.8)	61.5 (55.8–68.0)	62.0 (55.5–70.0)	65.0 (60.0–69.5)	0.170
Male, %	71 (52.6)	10 (50.0)	31 (50.0)	16 (55.2)	14 (58.3)	0.902
Body mass index, kg/m^2^	25.0 (23.0–27.0)	26.0 (24.3–26.9)	25.0 (23.0–27.0)	26.0 (24.0–27.5)	24.0 (22.0–27.0)	0.282
**Risk factors**						
Current smoking, %	61 (45.2)	8 (40.0)	26 (41.9)	15 (51.7)	12 (50.0)	0.753
Hypertension, %	97 (71.9)	13 (65.0)	46 (74.2)	21 (72.4)	17 (70.8)	0.895
Diabetes, %	45 (33.3)	3 (15.0)	25 (40.3)	7 (24.1)	10 (41.7)	0.102
Hyperlipidemia, %	73 (54.1)	8 (40.0)	38 (61.3)	14 (48.3)	13 (54.2)	0.360
Family history, %	38 (28.1)	9 (45.0)	13 (21.0)	8 (27.6)	8 (33.3)	0.196
**Lab examinations**						
Total triglyceride, mmol/L	1.6 (1.2–2.5)	2.0 (1.3–3.2)	1.7 (1.2–2.4)	1.5 (1.2–2.4)	1.4 (0.9–2.6)	0.316
Total cholesterol, mmol/L	5.1 (4.1–5.9)	5.2 (4.7–5.8)	5.1 (4.1–5.9)	5.2 (4.0–5.7)	4.7 (3.8–6.1)	0.680
LDL-C, mmol/L	3.2 (2.6–3.7)	3.0 (2.6–3.5)	3.2 (2.6–3.8)	3.2 (2.5–3.5)	3.3 (2.4–3.9)	0.903
Glycosylated hemoglobin, %	6.0 (5.6–6.8)	6.2 (5.7–6.9)	5.9 (5.6–6.7)	6.0 (5.5–6.6)	6.2 (5.6–7.7)	0.540
Creatinine, umol/L	63.0 (53.0–73.0)	58.0 (50.3–68.0)	66.0 (55.0–74.0)	65.0 (53.0–74.0)	61.5 (47.8–72.8)	0.216
hs-CRP, mg/L	1.6 (0.8–3.5)	0.9 (0.3–1.7)	1.6 (0.6–3.1)	2.0 (1.0–4.2)*	2.7 (1.2–4.5)*	0.005
White blood cell, *10^9^/L	6.2 (5.4–7.4)	6.1 (5.4–7.1)	6.2 (5.4–7.3)	6.4 (5.4–8.6)	5.7 (5.4–6.5)	0.610
Hemoglobin, g/L	132.0 (124.0–143.0)	132.0 (126.0–143.0)	134.0 (123.0–144.3)	130.0 (124.0–144.0)	133.5 (125.3–145.0)	0.915
Platelet, *10^9^/L	202.0 (161.0–245.0)	212.0 (175.8–229.0)	192.0 (145.5–233.5)	222.0 (168.5–256.0)	214.0 (155.8–251.8)	0.191
**Echocardiography**						
LVEF, %	66.0 (61.0–71.0)	65.5 (61.3–69.5)	66.0 (61.0–72.0)	67.0 (61.0–72.5)	63.5 (60.5–68.0)	0.754
LVEDD, mm	46.0 (44.0–49.0)	46.5 (44.0–49.0)	46.0 (44.0–49.0)	44.0 (42.0–46.0)	46.0 (44.0–48.8)	0.112
IVSD, mm	9.0 (8.0–10.0)	9.0 (8.1–9.8)	9.0 (8.0–10.0)	8.0 (7.0–9.5)	9.0 (8.0–10.0)	0.285

Data shown as median (interquartile range), or number (percent) as appropriate.

A *p*-value was calculated by the Kruskal-Wallis H test.

CAD, coronary artery disease; hs-CRP, hypersensitive-C-reactive protein; IVSD, interventricular septal thickness at diastole; LM, left main; LDL-C, low density lipoprotein cholesterol; LVEF, left ventricular ejection fraction; LVEDD, left ventricular end diastolic diameter.

* Indicated Bonferroni adjusted *p* < 0.05 compared to Non-CAD group.

**Table 2:** Medications and coronary intervention of the study population.

	Overall *N* = 135	Non-CAD *N* = 20	CAD *N* = 115
**Disease severity**			
Gensini score	20.0 (13.0–36.0)	7.5 (6.0–15.0)	23.0 (16.0–39.0)
**Disease location, %**			
Left main	–	–	3 (2.6)
Left anterior descending	–	–	90 (78.3)
Left circumflex	–	–	50 (43.5)
Right coronary artery	–	–	51 (44.3)
**Medication, %**			
Antiplatelet	130 (96.3)	15 (75.0)	115 (100.0)
Statins	125 (92.6)	13 (65.0)	112 (97.4)
ACEI/ARB	65 (48.1)	8 (40.0)	57 (49.6)
**Intervention, %**			
Any intervention	98 (72.6)	–	98 (85.2)
Stent implantation	87 (64.4)	–	87 (75.7)

Data shown as median (interquartile range), or number (percent) as appropriate.

ACEI, angiotensin-converting enzyme inhibitor; ARB, angiotensin receptor blocker; CAD, coronary artery disease.

After coronary angiography, CAD was identified in 115 patients (85.2%), with 45.9% of patients having 1-vessel disease, 21.5% of 2-vessel disease, and 17.8% of 3-vessel or LM disease (Table 1). These findings were consistent with an increased overall Gensini score (median, 20.0) (Table 2). Coronary artery stenosis was most likely to present in the left anterior descending artery (78.3%), followed by the right coronary artery (44.3%) and left circumflex artery (43.5%). Three patients (2.6%) had LM disease, which represented a severe type of CAD. Therefore, PCI was performed in 85.2% of CAD patients, with 75.7% having stent implantation (Table 2).

### Correlation of serum miR-183-5p with CAD severity

**Figure 1:**
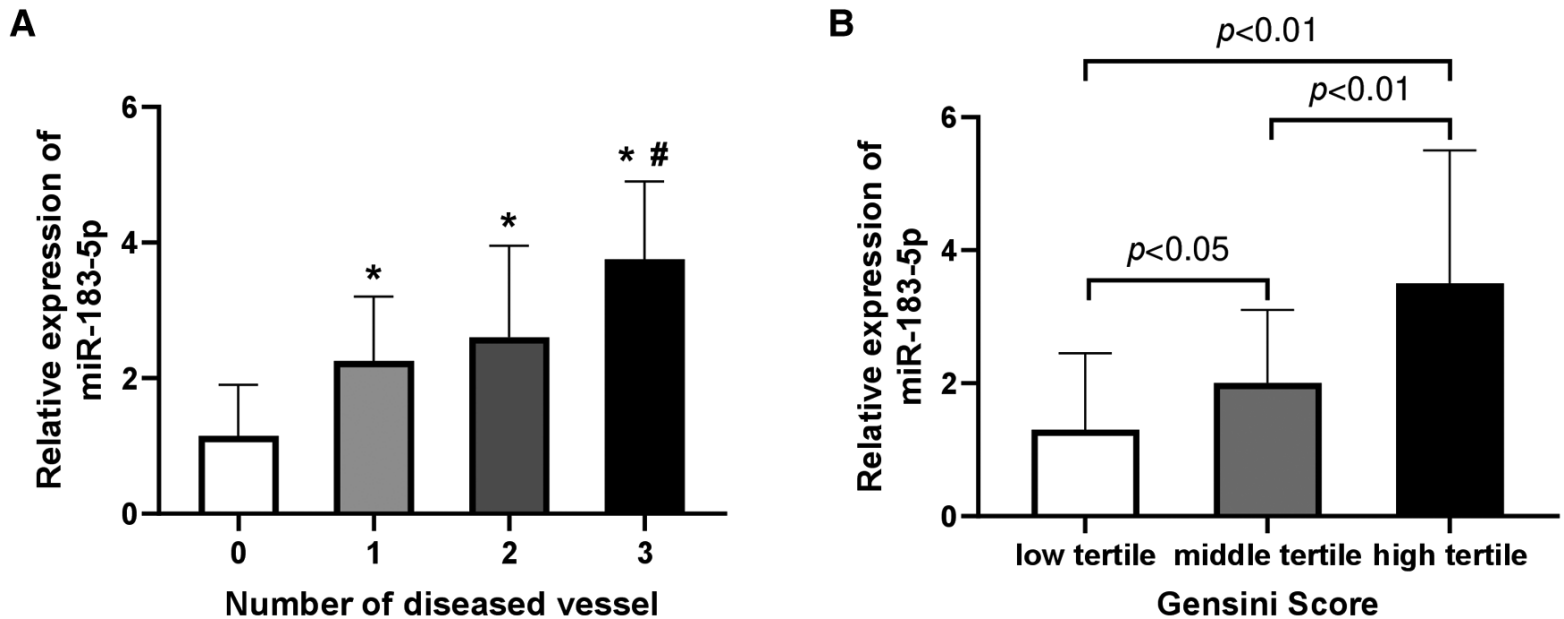
Relative expression of serum miR-183-5p levels according to number of diseased vessel (**A**) and gensini score tertiles (**B**). (**A**) *indicated Bonferroni adjusted *p* < 0.05 when compared to 0-vessel disease; #indicated Bonferroni adjusted *p* < 0.01 when compared to 1-vessel disease. (**B**) all *p*-values adjusted by Bonferroni correction.

Firstly, we found that serum miR-183-5p levels in any CAD subgroup were significantly increased than non-CAD patients (all adjusted *p* < 0.05) (Figure 1A). Among CAD subgroups, significantly increased miR-183-5p levels were observed in 3-vessel or LM than 1-vessel disease [3.75 [interquartile range (IQR): 2.45–4.90] vs. 2.25 (IQR: 1.45–3.20), adjusted *p* = 0.002] (Figure 1A). Moreover, a significant step-wise increased pattern between the Gensini score tertiles and miR-183-5p was showed [middle vs. low tertile: 2.10 (IQR: 1.60–2.80) vs. 1.20 (IQR: 0.80–2.55), adjusted *p* = 0.044], [high vs. middle tertile: 3.50 (IQR: 2.60–5.50) vs. 2.10 (IQR: 1.60–2.80), adjusted *p* < 0.01], [high vs. low tertile: 3.50 (IQR: 2.60–5.50) vs. 1.20 (IQR: 0.80–2.55), adjusted *p* < 0.01] (Figure 1B).

### CAD severity in tertile of serum miR-183-5p levels

**Figure 2:**
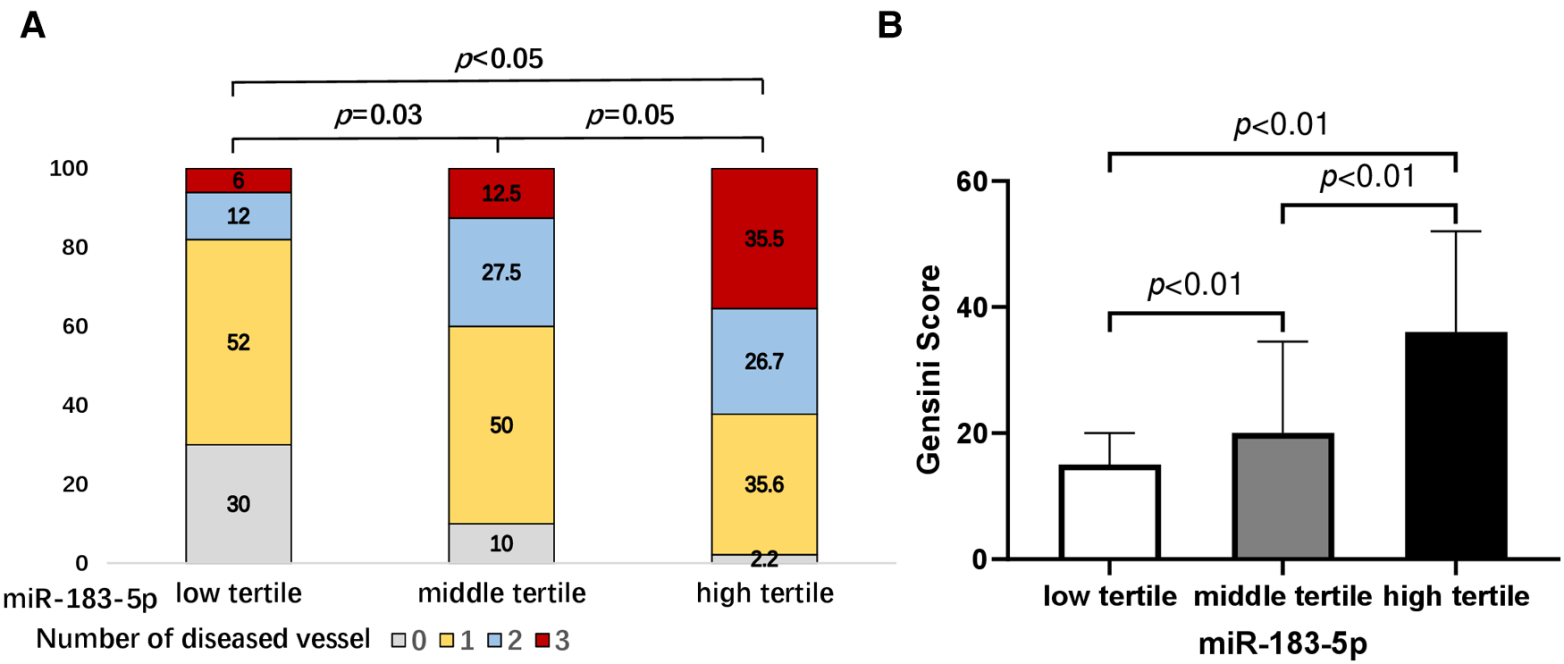
Proportions of number of diseased vessel (**A**) and gensini score (**B**) according to tertiles of serum miR-183-5p levels. All *p*-values adjusted by Bonferroni correction.

**Figure 3:**
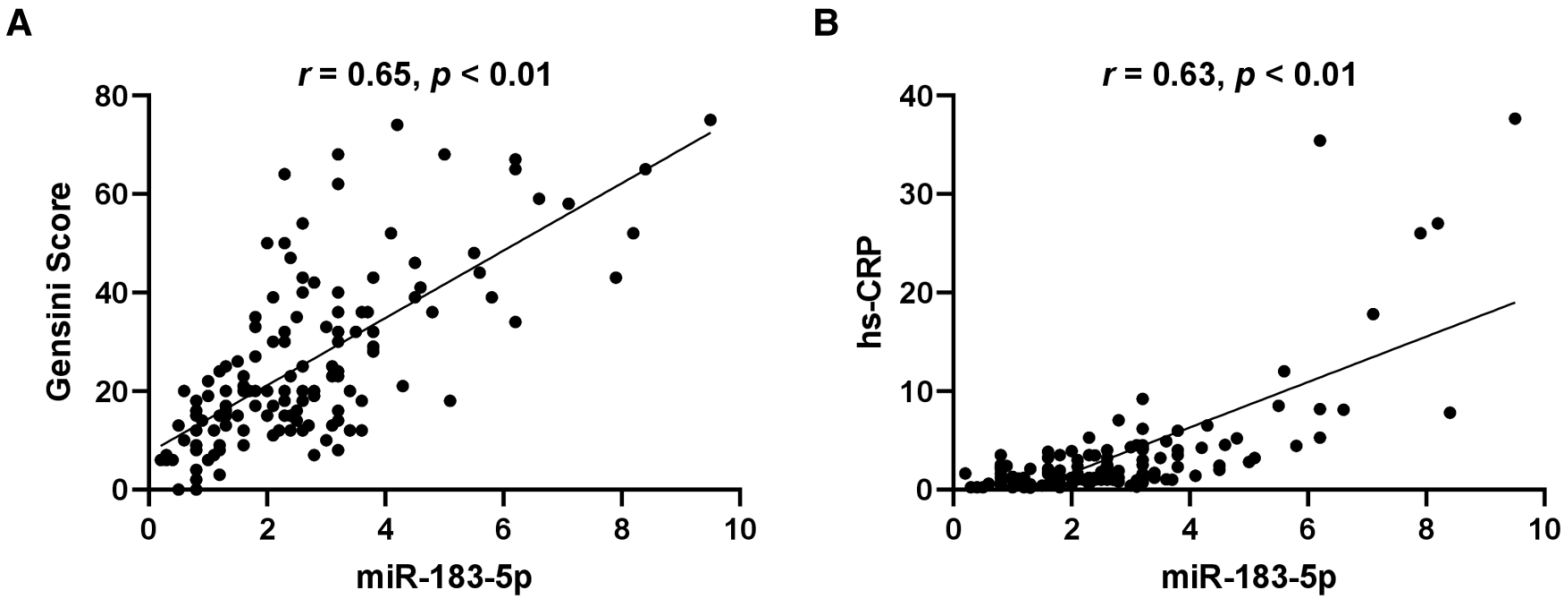
Correlation of serum miR-183-5p levels with gensini score (**A**) and hypersensitive-C-reactive protein (hs-CRP) (**B**).

Firstly, we found that, across tertiles of serum miR-183-5p levels, the proportion of multiple vessel disease increased, while single or none vessel disease decreased (all *p* < 0.05) (Figure 2A). Similarly, a significant step-wise increased pattern between miR-183-5p tertiles and the Gensini score was observed [middle vs. low tertile: 20.0 (IQR: 15.0–34.5) vs. 12.0 (IQR: 7.0–20.0), adjusted *p* = 0.003] [high vs. middle tertile: 36.0 (IQR: 25.0–52.0) vs. 20.0 (IQR: 15.0–34.5), adjusted *p* = 0.007] [high vs. middle tertile: 36.0 (IQR: 25.0–52.0) vs. 12.0 (IQR: 7.0–20.0), adjusted *p* < 0.01] (Figure 2B). Moreover, serum miR-183-5p levels were positively correlated with the Gensini score (*r* = 0.65, 95% CI: 0.54–0.74, *p* < 0.01) (Figure 3A) and hs-CRP (*r* = 0.63, 95% CI: 0.51–0.73, *p* < 0.01) (Figure 3B).

### Predictive value of serum miR-183-5p levels on CAD

**Figure 4:**
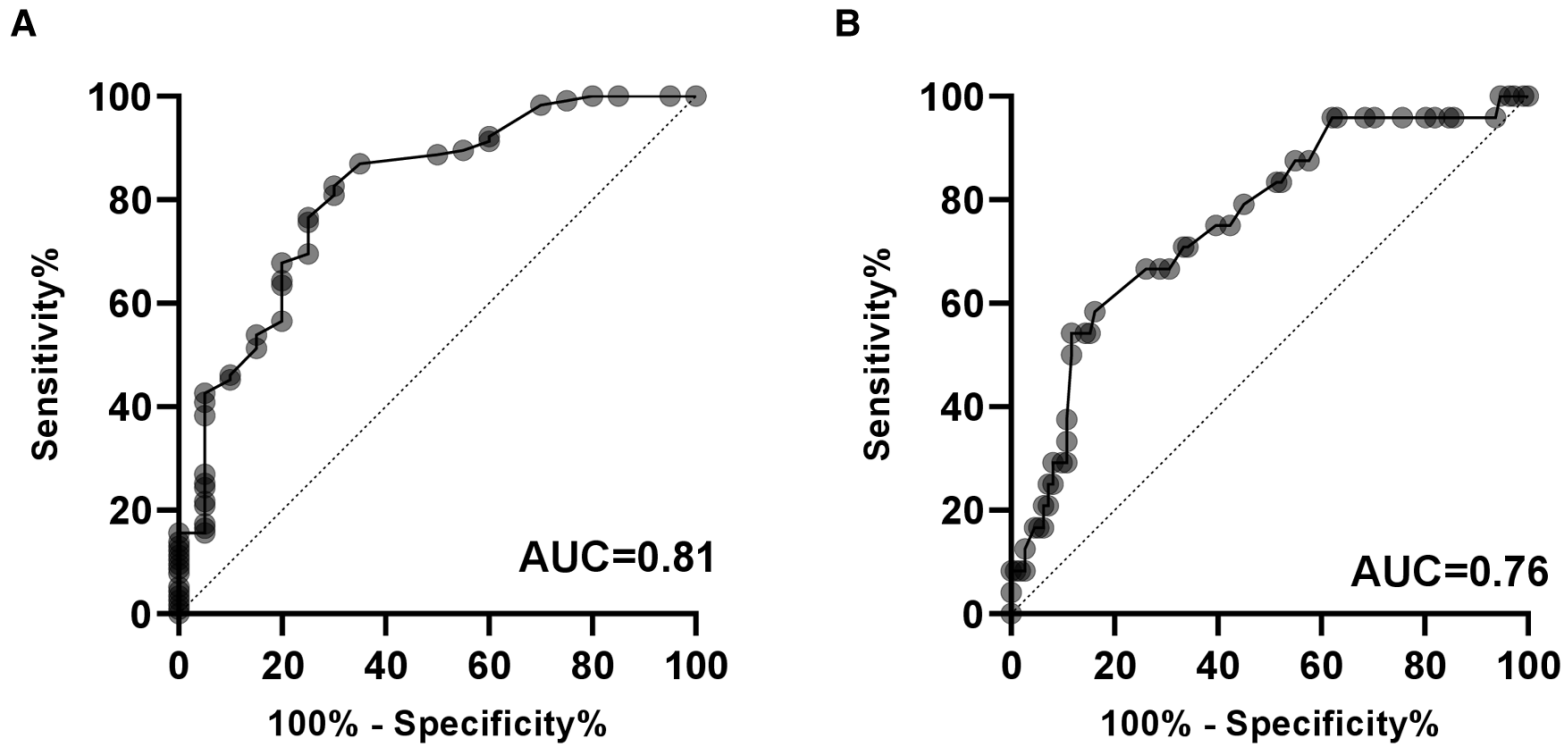
Diagnostic value of serum miR-183-5p levels on the presence of CAD (**A**) and severe CAD (i.e., 3-vessel or LM disease). (**B**) AUC, area under the receiver operating characteristic curve; CAD, coronary artery disease.

The predictive values of serum miR-183-5p levels on the presence of CAD (AUC 0.82, 95% CI 0.71–0.92, *p* < 0.01) and 3-vessel or LM disease (AUC 0.76, 95% CI 0.65–0.86, *p* < 0.01) were confirmed in the ROC curve analysis, respectively (Figure 4A). The optimal cut-off values of miR-183-5p predicting CAD presence and severity were 1.40 (sensitivity 82.6%, specificity 70.0%) and 3.65 (sensitivity 54.2%, specificity 88.3%), respectively (Figure 4B).

Furthermore, in multivariate analysis adjusting for age, sex, BMI, diabetes, hs-CRP, tertile of serum miR-183-5p levels showed increased predictive value on the presence of CAD (middle vs. low tertile, HR 3.81, 95% CI 1.07–13.56, *p *= 0.039; high vs. low tertile, HR 14.57, 95% CI 1.38–153.65, *p *= 0.026; *p* for trend = 0.024) and 3-vessel or LM disease (middle vs. low tertile, HR 2.11, 95% CI 0.46–9.70, *p *= 0.34; high vs. low tertile, HR 6.59, 95% CI 1.59–27.25, *p *= 0.009; *p* for trend = 0.020) (Table 3).

**Table 3:** Prediction of CAD presence and severe CAD of the study population.

Variables	Prediction of CAD presence						Prediction of 3-vessel or LM disease					
	Univariate			Multivariate			Univariate			Multivariate		
	HR	95% CI	*p*-value	HR	95% CI	*p*-value	HR	95% CI	*p*-value	HR	95% CI	*p*-value
Age	1.04	0.99–1.10	0.161	1.04	0.98–1.10	0.235	1.05	0.99–1.11	0.071	1.05	0.99–1.11	0.092
Male	1.13	0.44–2.92	0.801	1.06	0.37–3.05	0.914	1.33	0.54–3.24	0.535	1.29	0.47–3.54	0.618
BMI	0.93	0.77–1.12	0.448	0.93	0.76–1.14	0.476	0.88	0.73–1.05	0.160	0.85	0.70–1.05	0.124
Diabetes	3.26	0.90–11.78	0.071	3.52	0.91–13.61	0.068	–	–	–	–	–	–
hs-CRP	1.43	0.98–2.06	0.061	1.08	0.76–1.54	0.657	1.08	1.01–1.16	0.019	1.05	0.72–1.12	0.230
miR-183-5p			0.004*			0.024*			0.020*			0.020*
Low tertile	1.00	–	–	1.00	–	–	1.00	–	–	1.00	–	–
Middle tertile	3.86	1.17–12.77	0.027	3.81	1.07–13.56	0.039	2.24	0.50–10.00	0.291	2.11	0.46–9.70	0.34
High tertile	18.86	2.37–149.79	0.005	14.57	1.38–153.65	0.026	8.64	2.32–32.26	0.001	6.59	1.59–27.25	0.009

Variables in the left column included in the logistics regression analysis.

CAD, coronary artery disease; HR, hazard ratio; CI, confidence interval; BMI, body mass index; hs-CRP, hypersensitive-C-reactive protein.

*Indicated *p*-value for trend in the logistics regression analysis.

## Discussion

To the best of our knowledge, this is the first study investigating the relationship between serum miR-183-5p and stable CAD. The main findings of the present study included: (1) a significant step-wise increased pattern existed between the Gensini score tertiles and serum miR-183-5p levels; (2) serum miR-183-5p levels were positively correlated with the Gensini score and hs-CRP; (3) serum miR-183-5p levels could predict CAD presence and severity, with optimal cut-off values of 1.40 (sensitivity 82.6%, specificity 70.0%) and 3.65 (sensitivity 54.2%, specificity 88.3%), respectively; (4) the predictive value of serum miR-183-5p levels on CAD presence and severity were confirmed in multivariable analysis.

MiR-183 belongs to the miR-183 cluster, consisting of three microRNAs: miR-183, miR-96 and, miR-182. These homologous microRNAs are highly co-expressed in the murine retina, and their chromosomal loci are quite close (18). Increased miR-183 cluster members have been found in autoimmune diseases, neuronal and psychiatric disorders, and various malignancies (19). MiR-183 was identified in 2003 by cloning from the human Saos-2 cell line and mouse tissues (20). MiR-183-5p is known to overexpress in the peripheral blood mononuclear cells (PBMCs) (21) of breast cancer, in the tumor tissue of breast cancer (9), primary nasopharyngeal carcinoma (11), and hepatocellular carcinoma (10). In addition, elevated miR-183-5p is observed in PBMCs from patients with systemic lupus erythematosus (22), and in plasma-derived exosomes from patients with intestinal Behçet's syndrome (23).

The main underlying pathophysiology of CAD is atherosclerosis and/or thrombosis, which is a chronic inflammatory disease. Recently, Meerson et al. found that, compared to healthy individuals, plasma miR-183-5p levels were significantly increased in women with early diabetes (an important risk factor of atherosclerosis) (24). Meanwhile, overexpression of miR-183-5p was observed in the serum of patients with carotid atherosclerosis (12, 13), and positively correlated with carotid intima-media thickness (13). Similarly, elevated miR-183 levels were detected in the plasma exosome from patients with MI than in healthy individuals, which positively correlated with the degree of myocardial injury (14). Recently, Liu found that exosomal miR-183-5p from epicardial adipose tissue of patients with CAD were increased compared with those without CAD (25). Interestingly, Tong et al. found that plasma miR-183-5p levels were novel diagnostic markers only for NSTEMI, but not for STEMI (15). These discrepancies probably resulted from different pathophysiology of these two CAD subtypes, but also different sensitivity and specificity of miR-183-5p detecting methods (i.e., RNA-seq and qPCR).

Circulating miR-183-5p might be a promising marker of carotid atherosclerosis and ACS. However, less is known about its level in stable CAD patients. To fill the gap, we performed this cross-sectional study to investigate the relationship between serum miR-183-5p levels and CAD presence and severity. We found that serum miR-183-5p levels were increased in CAD patients with different severities than in non-CAD control. Serum miR-183-5p levels were positively correlated with the Gensini score, and hs-CRP, respectively. Interestingly, in patients with carotid atherosclerosis, serum miR-183-5p levels were positively correlated with CRP (13) and ox-LDL (12), which are well-known causes of atherosclerosis. Importantly, serum miR-183-5p levels had a relatively high power to diagnose the presence of CAD (AUC 0.82) and 3-vessel or LM disease (AUC 0.76). The optimal cut-off values of miR-183-5p predicting CAD presence and severity were 1.40 and 3.65, respectively, which were higher than its cut-off point predicting the presence of carotid atherosclerosis (0.91) in the study of Sun et al. (13). Moreover, this predictive value of serum miR-183-5p level was independent of age, sex, BMI, diabetes, and hs-CRP, which are well-established risk factors of CAD. Therefore, circulating miR-183-5p level is a marker of all categories of CAD presence, which could be used for early diagnosis of CAD; Meanwhile, serum miR-183-5p level could dynamically reflect CAD severity; More importantly, miR-183-5p might be a therapeutic target after fully elucidating the underlying mechanism between miR-183-5p and atherosclerosis/thrombosis.

Although the detrimental effects of miR-183-5p on vasculature have been reported, its exact mechanism leading to atherosclerosis is not fully understood. Zhang et al. showed that down-regulation of miR-183-5p in ox-LDL-treated human umbilical vascular endothelial cells could attenuate cell injury and inflammation by upregulation of insulin receptor substrate 1 (26). In subarachnoid hemorrhage rats, bone marrow mesenchymal stem cell-derived extracellular vesicles could alleviate endothelial dysfunction by regulating the KLF3-AS1/miR-183-5p/TCF7L2 signaling axis (27). In addition, Sun et al. found that overexpression of miR-183-5p accelerated the proliferation and migration of vascular smooth muscle cells (VSMCs) (13). Similarly, Fan et al. found, in VSMCs treated with ox-LDL, miR-183-5p was overexpressed, which may down-regulate FOXO1, leading to proliferation/apoptosis imbalance in VSMCs (12). Importantly, miR-183-5p might intervene the initiation and development of atherosclerosis by impacting not only vascular endothelial and VSMCs, but macrophages. In bone marrow-derived macrophages (BMDMs) transfected with a miR-183 inhibitor, the foam-cell formation was reduced, and cholesterol efflux increased (28). Furthermore, in BMDMs subjected to ox-LDL, miR-183 knockdown decreased the M1/M2 ratio with attenuated NF-_k_B activation, via targeting NR4A2 (28). However, exosomal miR-183-5p derived from bone marrow mesenchymal stem cell could protect ischemia/reperfusion injury in cardiomyocytes by targeting FOXO1 (29) or voltage-dependent anion channel 1 (30). These findings illustrated that miR-183-5p could potentially have multiple effects on the cardiovascular system.

The present study has several limitations. (1) study population was restricted to those without ASCVD, which may limit the extrapolation of the study results to a broader population; (2) number of the study population was relatively small, precluding us from finding a statistical difference of miR-183-5p between CAD patients with different numbers of diseased vessels; (3) given the cross-sectional design, a causal relation between miR-183-5p and CAD cannot be determined; (4) although the multivariate analysis was performed, the residual confounders affecting the correlation between miR-183-5p and CAD cannot be excluded; (5) CAD severity was judged by experienced physician visually, which may be improved using intracoronary imaging and functional examination.

## Conclusions

In the population with chest pain who have no history of established ASCVD, serum miR-183-5p levels are independently and positively correlated with stable CAD presence and severity. Further studies are needed to elucidate the physiopathologic mechanism between miR-183-5p and atherosclerosis, as well as the outcome predictive value of miR-183-5p.

## Data Availability

The raw data supporting the conclusions of this article will be made available by the authors, without undue reservation.
